# Role of biomarkers in early infectious complications after lung transplantation

**DOI:** 10.1371/journal.pone.0180202

**Published:** 2017-07-13

**Authors:** Borja Suberviola, Luzdivina Rellan, Jordi Riera, Reyes Iranzo, Ascension Garcia Campos, Juan Carlos Robles, Rosario Vicente, Eduardo Miñambres, Miguel Santibanez

**Affiliations:** 1 Critical Care Department, Hospital Universitario Marqués de Valdecilla – IDIVAL, Santander, Spain; 2 Department of Anesthesiology, Complexo Hospitalario Universitario A Coruna, A Coruna, Spain; 3 Critical Care Department, Hospital Vall d'Hebron, Barcelona, Spain; 4 Department of Anesthesiology, Hospital Universitario Puerta de Hierro Majadahonda, Madrid, Spain; 5 Department of Anesthesiology, Hospital Universitario 12 de Octubre, Madrid, Spain; 6 Transplant Coordination Unit, Hospital Universitario Reina Sofia, Cordoba, Spain; 7 Department of Anesthesiology, Hospital Universitario y Politécnico de La Fe, Valencia, Spain; 8 Critical Care Department - Transplant Coordination Unit, Hospital Universitario Marques de Valdecilla – IDIVAL, Santander, Spain; 9 Nurse School, Universidad de Cantabria – IDIVAL, Santander, Spain; University of Toledo, UNITED STATES

## Abstract

**Background:**

Infections and primary graft dysfunction are devastating complications in the immediate postoperative period following lung transplantation. Nowadays, reliable diagnostic tools are not available. Biomarkers could improve early infection diagnosis.

**Methods:**

Multicentre prospective observational study that included all centres authorized to perform lung transplantation in Spain. Lung infection and/or primary graft dysfunction presentation during study period (first postoperative week) was determined. Biomarkers were measured on ICU admission and daily till ICU discharge or for the following 6 consecutive postoperative days.

**Results:**

We included 233 patients. Median PCT levels were significantly lower in patients with no infection than in patients with Infection on all follow up days. PCT levels were similar for PGD grades 1 and 2 and increased significantly in grade 3. CRP levels were similar in all groups, and no significant differences were observed at any study time point. In the absence of PGD grade 3, PCT levels above median (0.50 ng/ml on admission or 1.17 ng/ml on day 1) were significantly associated with more than two- and three-fold increase in the risk of infection (adjusted Odds Ratio 2.37, 95% confidence interval 1.06 to 5.30 and 3.44, 95% confidence interval 1.52 to 7.78, respectively).

**Conclusions:**

In the absence of severe primary graft dysfunction, procalcitonin can be useful in detecting infections during the first postoperative week. PGD grade 3 significantly increases PCT levels and interferes with the capacity of PCT as a marker of infection. PCT was superior to CRP in the diagnosis of infection during the study period.

## Introduction

Infection and primary graft dysfunction (PGD) are the most common and devastating complications in the immediate postoperative period following lung transplantation (LT) [1]. According to the registry of the International Society for Heart and Lung Transplantation, in a total of 45,542 lung transplants the major reported causes of death within the first 30 days after transplantation were PGD (24.3%) and non-CMV infections (19.3%) [2]. Direct contact of the allograft with the environment, impaired clearance mechanisms caused by allograft denervation and profound immunosuppression, especially in the first postoperative days, explain this high vulnerability of LT recipients to infection.

Early diagnosis of infectious complications after LT is imperative. This allows prompt initiation of antimicrobial therapy and adjustment of immunosuppressant therapy, and can prevent infection-related morbidity and mortality [3,4]. However, some characteristics inherent to lung transplantation make this early diagnosis difficult. The need for higher immunosuppression as compared with other allografts can mask common infectious symptoms like fever and delay infection recognition. In addition, lung infection and PGD share respiratory failure and lung infiltrates on chest X-ray as the most common symptoms making differential diagnosis complicated [5]. As diagnostic errors in these vulnerable patients can have harmful consequences, reliable diagnostic tools are desirable.

Various studies have demonstrated that procalcitonin (PCT) is a useful diagnostic marker of infection in different critical patient groups [6]. In transplant recipients, PCT has been shown on several occasions to be a valuable parameter for detecting infection in liver and heart transplant recipients [7–11]. However, few data have been published on PCT after lung transplantation, and none on PGD. Only 2 studies, 1 of them published by our group, have evaluated the benefit of PCT as a diagnostic marker of infection. Although the findings were promising, the quality of the studies was marred by the small sample size. Therefore, the role of C reactive protein (CRP) in this context remains unclear.

The aims of the study were to determine how serial determination of PCT and CRP can improve the diagnosis of early infectious complications after lung transplantation, and to determine whether their predictive accuracy is affected by the presence of PGD.

## Methods

### Study design and setting

Full names of all hospitals and approving IRBs/ethics committees are: University Hospital Reina Sofia (Cordoba): Comité de Ética de la Investigación de Córdoba. University Hospital Vall’dHebron (Barcelona): Comité Ético de Investigación Clínica del Hospital Universitario Vall d’Hebron. University Hospital Puerta de Hierro (Madrid): Comite de evaluación de investigación con medicamentos CEIm. University Hospital La Fe (Valencia): Comité Etico de Investigación Biomedica. University Hospital A Coruña (La Coruña): Fundación Profesor Novoa Santos. University Hospital 12 de Octubre (Madrid): Comité de Etica Hospital 12 de Octubre. University Hospital Marques de Valdecilla (Santander): Comité Etico de Investigacion Clinica de Cantabria.

We conducted a multicentre prospective observational study between September 2014 and September 2015. The study included all 7 centres authorized to perform lung transplantation in Spain. All data regarding lung transplantation are available on the Spanish National Transplant Organization website [12].

The study period included the first 7 postoperative days after lung transplantation. Clinical and demographic characteristics of all patients, including age, gender, type of transplant (single or bilateral), need for extracorporeal membrane oxygenation (prior to transplantation, during or/and after surgery), cold ischemia time, infection diagnosis, source of infection, length of ICU and hospital stay and ICU and hospital mortality were recorded. Patients were divided into 2 groups depending on the presence of infectious complications during the study period (day of ICU admission and the following 6 days after lung transplantation). Lung Infection [13] and primary graft dysfunction [14] were defined according to the International Society for Heart and Lung Transplantation definitions, and ordinal categorized according to severity. Severe PGD was defined as PGD grade 3.

### Methods of measurement

Biomarkers were measured on ICU admission (day 0) and daily till ICU discharge or for the following 6 consecutive postoperative days, as appropriate. Except for day 0, blood samples were obtained at 7 am. In all cases, biomarkers were assessed in real time.

Serum PCT was measured with a time-resolved amplified cryptate emission technology assay (Kryptor PCT; Brahms, Hennigsdorf, Germany). This assay is based on a polyclonal antibody against calcitonin and on a monoclonal antibody against katacalcin. Antibodies bind to the calcitonin and katacalcin sequence of precursor molecules. This assay has an optimised functional sensitivity of 0.06 mcg/L.

CRP concentrations were measured by immunoturbidimetry using a modular analyser (Roche Diagnostics, Meylan, France).

### Statistical analysis

Categorical and discrete variables were expressed as counts (percentage) and continuous variables as mean ± standard deviation or median and 25–75 percentile (Interquartile Range) in case of not normally distributed variables such as PCT and CRP levels. Statistical differences between groups were assessed by the chi-square test, using Yates’ correction or Fisher’s exact test for categorical variables when appropriate. U Mann Whitney and Kruskall Wallis tests were used for PCT and CRP levels and other not normally distributed variables, and the student’s t test was used for the remaining continuous variables.

To compare the accuracy of PCT levels to predict the risk of suffering ‘Infection’ for each day of follow-up, receiver operating characteristic (ROC) curves were constructed and the areas under the curve (AUC) with their 95% confidence intervals (95% CI) were determined.

To estimate the strength of association, the PCT values were divided into dichotomous variables using the median split method, and adjusted odds ratios (OR) with their 95% CI for the risk of suffering ‘Infection’ were calculated using unconditional logistic regression. The following potential confounders were pre-established for inclusion in the models: sex, age (as a continuous variable), necessity of post-operative Extracorporeal Membrane Oxygenation (ECMO) (yes/no), creatinine levels (as a continuous variable), type of lung transplant (single or double-lung transplantation) and transplant centre (hospital). Analysis was stratified according to the presence or absence of PGD Grade 3.

The level of statistical significance was set at 0.05, and all tests were two-tailed. We used the SPSS statistical software package 22.0 (SPSS, Inc., Chicago, Ill) for all statistical analyses.

All donation permissions were obtained according to Spanish donor laws. This includes the need of specific permission signed by patient relatives. In particular, in this study we have not used donated tissue/organs from any vulnerable populations. The hospital’s ethics committees of all participant centres accepted the study and informed consent was obtained from participants for inclusion in this study and for the use of their clinical samples and data in research.

## Results

### Demographic and clinical characteristics

Two hundred and thirty three consecutive patients (148 men, mean age 52.97 ± 11.86 years) underwent single (*n* = 108, 46.4%) or bilateral (*n* = 125, 53.6%) LT for pulmonary fibrosis (44.6%), emphysema (20.6%), COPD (15%) and other causes (19.8%). Inclusion by hospital was as follows: University Hospital “Marques de Valdecilla” (Santander) 41 patients (17.6%), University Hospital “A Coruña” (La Coruña) 38 patients (16.3%), University Hospital “Vall d’Hebron” (Barcelona) 38 patients (16.3%), University Hospital “Puerta de Hierro” (Madrid) 35 patients (15%), University Hospital “Doce de Octubre” (Madrid) 29 patients (12.4%), University Hospital “Reina Sofía” (Córdoba) 29 patients (12.4%) and University Hospital “La Fé” (Valencia) 23 patients (9.9%). Baseline characteristics are shown in Table 1.

**Table 1:** Baseline characteristics of the study population.

	Overall population	No infection	Infection in trasplant recipient	*p value*
	N = 233	N = 181	N = 52	
Age (years), median (IQR)	57 (48–61)	57 (48–61)	55.5 (48.5–61)	*0*.*961*
Male sex, n (%)	148 (63.5)	110 (60.8)	38 (73.1)	*0*.*104*
Disease that origins transplant, n (%)				*0*.*845*
Pulmonary fibrosis	104 (44.6)	81 (77.9)	23 (22.1)	
Emphysema	48 (20.6)	35 (72.9)	13 (27.1)	
COPD	35 (15.0)	28 (80.0)	7 (20.0)	
Cystic fibrosis	18 (7.7)	14 (77.8)	4 (22.2)	
Pulmonary hypertension	9 (3.9)	8 (88.9)	1 (11.1)	
Others	15 (6.4)	11 (73.3)	4 (26.7)	
Donor infection, n (%)	57 (24.5)	38 (21.0)	19 (36.5)	*0*.*02*
Single lung transplant, n (%)	108 (46.4)	93 (51.4)	15 (28.8)	*0*.*004*
Ischemia 1^st^ lung (hours), median (IQR)	4.4 (4.0–5.2)	4.3 (3.4–5.0)	5.2 (4.4–5.9)	*0*.*09*
Ischemia 2^nd^ lung (hours), median (IQR)	7.0 (6.0–7.4)	6.4 (5.3–7.1)	7.6 (6.7–9.1)	*0*.*003*
Intraoperative ECMO, n (%)	36 (15.5)	29 (16.0)	7 (13.5)	*0*.*653*
Postoperative ECMO, n (%)	13 (5.6)	7 (3.9)	6 (11.5)	*0*.*034*
Primary Graft Dysfunction (PGD)				*0*.*018*
No PGD	139 (59.7)	100 (55.2)	39 (75)	
PGD Grade 1, n (%)	44 (18.9)	41 (22.7)	3 (5.8)	
PGD Grade 2, n (%)	22 (9.4)	16 (8.8)	6 (11.5)	
PGD Grade 3, n (%)	28 (12.0)	24 (13.3)	4 (7.7)	
ICU stay (days), median (IQR)	6.9 (4.0–16.4)	5.9 (4.0–13.9)	12.9 (6.2–29.7)	*0*.*19*
Hospital stay (days), median (IQR)	32.9 (23.9–52.4)	29.9 (23.4–43.9)	46.9 (29.9–73.6)	*0*.*37*
ICU mortality, n (%)	14 (6.0)	7 (3.9)	7 (13.5)	*0*.*01*
Hospital mortality, n (%)	18 (7.7)	11 (6.1)	7 (13.5)	*0*.*13*

IQR = Interquartile Range.

With respect to overall complications, 52 patients developed infection and 28 PGD. S1 Table shows a cross-table of each complication. In total, 157 patients presented a normal postoperative course without infection or PGD, 48 patients (20.6%) developed only ‘Infection’, 24 patients (10.3%) developed only ‘PGD grade 3’ and 4 patients (1.7%) developed ‘PGD grade 3’ followed by ‘Infection’ during follow-up.

Mean time between end of transplant surgery and diagnosis of infection was 2.23 ± 2.25 days (S2 Table). Infections were distributed as follows; ventilator-associated tracheobronchitis (48%), ventilator-associated pneumonia (35%) and non-ventilator-associated pneumonia (17%). The main organisms involved were *Pseudomonas aeruginosa* (43.2%), *Methicillin-susceptible Staphylococcus aureus* (15.5%), *Serratia Marcescens* (8.4%) and *Methicillin-resistant Staphylococcus aureus* (7.6%). Patients who developed infectious complications more frequently underwent bilateral LT, more frequently needed postoperative ECMO support and developed significantly higher grades of PGD (Table 1).

No cases of early rejection or viral infection were observed during the study period. Eighteen patients died after a median interval of 13 (range 2 to 112) days. The median length of ICU stay was 7 (range 4 to 16) days for the overall population and 13 (range 6 to 29) days for patients who developed infection.

### Biomarker kinetic profiles and postoperative complications

Valid cases and descriptive statistics for PCT and CRP levels for each day of follow-up with respect to Infection and PGD grade 3 are presented in S3 Table. PCT and CRP levels were not normally distributed, distribution was very asymmetrical, and so medians and P5 and P75 percentile are presented as measures of central tendency and dispersion in Table 2. Both the PCT and CRP biomarkers presented similar kinetics in all patients during the study period, with an initial increase in levels, a plasma peak recorded in the first 48 hours, and a progressive decline over the following days (Table 2).

**Table 2:** Median levels for PCT and CRP levels for each day of follow-up, in ‘Infection’ and ‘primary graft dysfunction (PGD) grade 3’ specifically.

	Overall	No Infection	Infection in trasplant recipient		No PGD Grade 3	PGD Grade 3	
	N = 233	N = 181	N = 52		N = 205	N = 28	
**PCT LEVELS**	**Median**	**Median**	**Median**	***P value***	**Median**	**Median**	***P value***
**Admiss**	0.50	0.36	2.00	*0*.*000*	0.47	4.57	*0*.*000*
**Day 1**	1.17	1.01	3.83	*0*.*000*	1.07	4.90	*0*.*000*
**Day 2**	1.14	0.94	2.73	*0*.*000*	1.01	2.61	*0*.*006*
**Day 3**	0.70	0.55	1.60	*0*.*000*	0.69	1.32	*0*.*402*
**Day 4**	0.56	0.50	0.78	*0*.*002*	0.54	1.11	*0*.*105*
**Day 5**	0.28	0.20	0.40	*0*.*006*	0.28	0.48	*0*.*680*
**Day 6**	0.20	0.20	0.30	*0*.*027*	0.20	0.39	*0*.*447*
**CRP LEVELS**	**Median**	**Median**	**Median**	***P value***	**Median**	**Median**	***P value***
**Admiss**	4.29	4.29	4.34	*0*.*635*	4.00	5.64	*0*.*351*
**Day 1**	12.32	12.25	12.90	*0*.*539*	12.90	11.40	*0*.*722*
**Day 2**	10.10	9.80	10.90	*0*.*209*	10.00	12.50	*0*.*346*
**Day 3**	5.97	5.76	6.60	*0*.*153*	5.72	8.46	*0*.*345*
**Day 4**	4.10	3.80	4.90	*0*.*090*	3.99	5.70	*0*.*107*
**Day 5**	3.01	2.55	4.10	*0*.*086*	2.62	5.40	*0*.*034*
**Day 6**	2.30	2.42	2.16	*0*.*451*	2.10	5.54	*0*.*068*

#### Primary graft dysfunction

**Fig 1:**
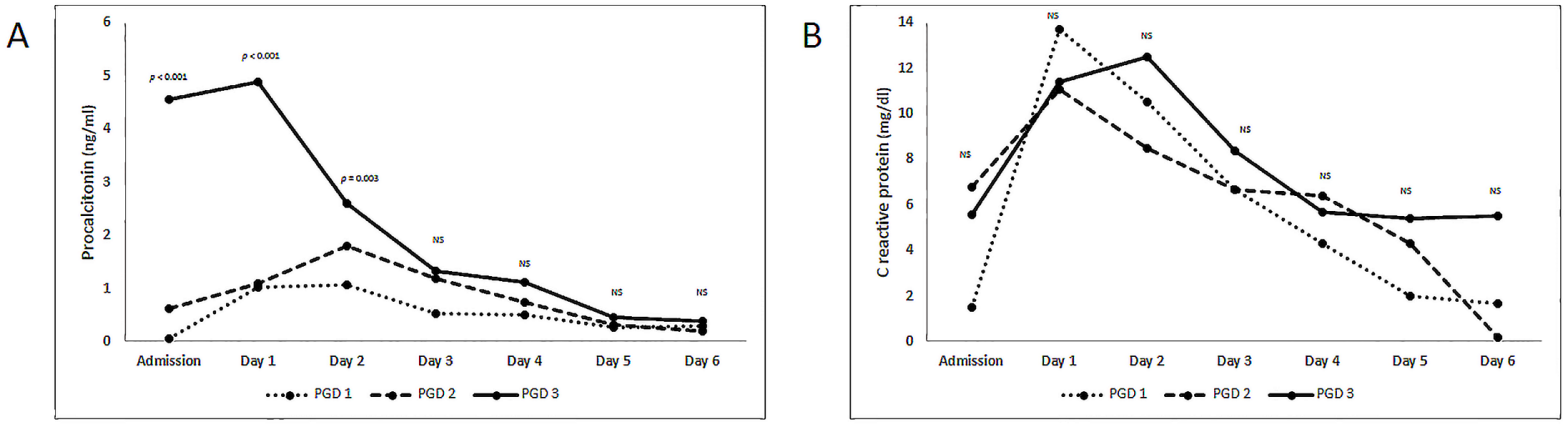
Biomarker levels and primary graft dysfunction.

With respect to PGD grades, 45 patients (19.3%) presented PGD 1, 25 (10.7%) grade 2 and 28 (12%) PGD grade 3. PCT plasma levels were similar for PGD grades 1 and 2 and increased significantly in the group of patients with PGD grade 3. CRP levels were similar in all groups, and no significant differences were observed at any study time point (Fig 1).

PCT levels were significantly higher in the first 3 postoperative days (day 0 to day 2) in the ‘PGD grade 3’ group vs. the rest of the study population, with a peak PCT plasma level observed on day 1 (mean: 4.90 ng/ml). In the case of CRP, significant differences were observed only on postoperative day 5 (p = 0.034) (Table 2).

#### Infection

**Fig 2:**
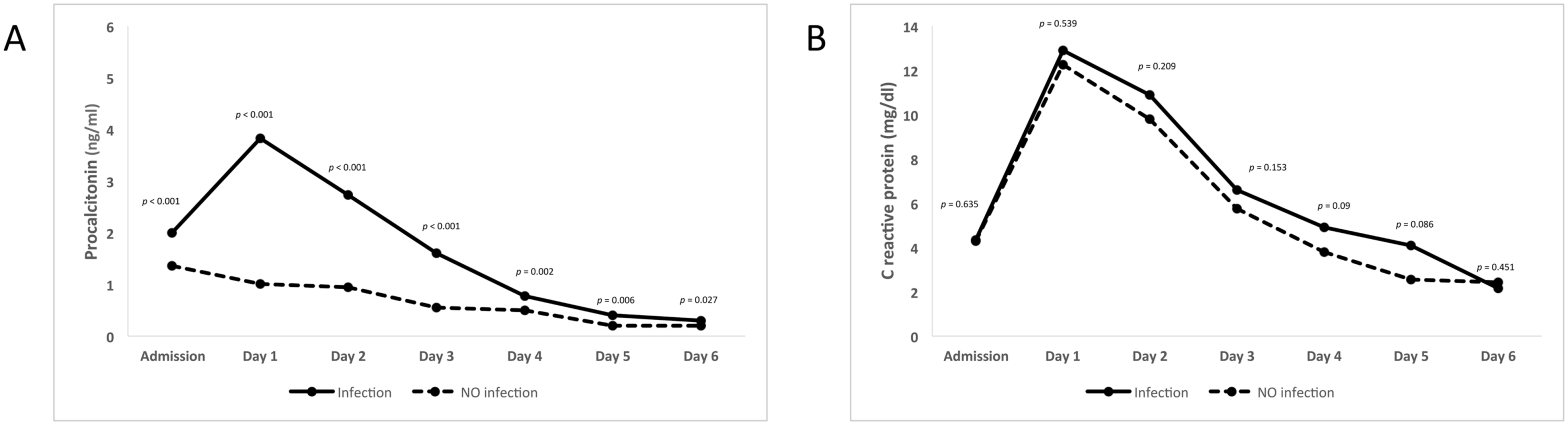
Biomarker levels and postoperative infection.

Median PCT levels were significantly lower in patients with no infection than in patients with ‘Infection’ on all follow up days. The greatest difference occurred between the day of admission and day 3 (p<0.001). PCT plasma levels peaked on day 1 (median: 3.83 ng/ml). In the case of CRP, the kinetic profile was similar in both groups, and no significant differences were observed with respect to the presence of infectious complications during the study period (Table 2 and Fig 2).

### Diagnostic accuracy and strength of association for PCT with respect to infection

Table 3 shows the diagnostic accuracy of PCT values for detecting ‘infection’ for each day of follow-up. According to the AUC levels, the diagnostic accuracy of PCT to determine the presence of infection was low (around 0.70) and increased slightly when focused exclusively on patients without primary graft dysfunction. For CRP, AUC levels were lower than those observed in PCT.

**Table 3:** Predictive accuracy of PCT values for each day of follow-up for the risk of infection, stratified by existence of primary graft dysfunction (PGD) grade 3.

PCT LEVELS	All Patients (N = 233)			Without PGD Grade 3 (N = 205)			With PGD Grade 3 (N = 28)		
	AUC	95%	CI	AUC	95%	CI	AUC	95%	CI
**Admiss**	0.70	0.62	0.78	0.73	0.64	0.82	0.69	0.32	1.00
**Day 1**	0.70	0.61	0.79	0.73	0.64	0.82	0.57	0.24	0.90
**Day 2**	0.70	0.61	0.80	0.73	0.63	0.83	0.50	0.14	0.86
**Day 3**	0.71	0.61	0.80	0.73	0.64	0.83	0.38	0.08	0.67
**Day 4**	0.67	0.57	0.77	0.70	0.60	0.80	--	--	--
**Day 5**	0.65	0.56	0.75	0.67	0.57	0.77	--	--	--
**Day 6**	0.63	0.52	0.74	0.66	0.54	0.77	--	--	--

AUC = Area Under the Curve; 95%CI = 95% Confidence Intervals

PCT levels were dichotomously categorized using the median split method in order to evaluate their strength of association with respect to infection (Table 4). During the first 5 postoperative days (day 0 to day 4), PCT levels above median value were statistically associated with a higher risk of infection. Thus, PCT levels above 0.50 ng/ml on ICU admission (day 0) or 1.17 ng/ml on postoperative day 1 were associated with a two- and three-fold increase in the risk of infection: OR 2.65; 95%CI (1.34–5.25) and OR 3.21; 95%CI (1.62–6.39), respectively. These associations remain significant after adjusting for sex, age (as continuous variable), need for postoperative Extracorporeal Membrane Oxygenation use (yes/no), creatinine levels (as a continuous variable), type of lung transplant (single or double-lung transplantation) and trasplant centre (hospital). However, after stratifying by primary graft dysfunction, these associations disappeared with respect to the group of patients with PGD 3 and increased in the No PGD 3 group. In the absence of PGD 3, therefore, previously described cut-off levels for day 0 and day 1 were significantly associated with a three- and four-fold increase in the risk of infection: OR 3.06; 95%CI (1.49–6.25), OR 4.10; 95%CI (1.99–8.46), respectively.

**Table 4:** Crude and adjusted odds ratios (OR) for PCT levels (according to median) in relation to existence or not of infection.

		**All Patients (N = 233)**							
		**Infection**							
**PCT LEVELS**	**Cut-off point**^**a**^	**NO (N)**	**YES (N)**	**ORc**^**b**^	**(95%**	**CI)**	**ORa**^**c**^	**(95%**	**CI)**
Admiss	<= .50	97	15	1.00	--		1.00	--	
	.51+	78	32	2.65	1.34	5.25	2.78	1.10	7.05
Day 1	< = 1.17	99	14	1.00	--		1.00	--	
	1.18+	77	35	3.21	1.62	6.39	3.07	1.39	6.82
Day 2	< = 1.14	86	13	1.00	--		1.00	--	
	1.15+	63	35	3.68	1.80	7.51	3.24	1.46	7.20
Day 3	< = 0.695	67	11	1.00	--		1.00	--	
	0.696+	48	30	3.81	1.74	8.34	3.73	1.49	9.36
Day 4	< = 0.56	64	11	1.00	--		1.00	--	
	0.57+	46	27	3.42	1.54	7.58	3.29	1.31	8.30
Day 5	< = 0.28	57	13	1.00	--		1.00	--	
	0.29+	47	23	2.15	0.98	4.69	2.33	0.92	5.92
Day 6	< = 0.20	46	12	1.00	--		1.00	--	
	0.21+	38	19	1.92	0.83	4.44	2.10	0.69	6.44
		**Without PGD Grade 3 (N = 205)**							
		**Infection**							
**PCT LEVELS**	**Cut-off point**	**NO (N)**	**YES (N)**	**ORc**^**b**^	**(95%**	**CI)**	**ORa**^**c**^	**(95%**	**CI)**
Admiss	<= .50	90	14	1.00	--		1.00	--	
	.51+	61	29	3.06	1.49	6.25	2.85	1.07	7.59
Day 1	< = 1.17	95	13	1.00	--		1.00	--	
	1.18+	57	32	4.10	1.99	8.46	3.90	1.64	9.23
Day 2	< = 1.14	80	12	1.00	--		1.00	--	
	1.15+	51	32	4.18	1.97	8.86	3.76	1.57	9.01
Day 3	< = 0.695	63	10	1.00	--		1.00	--	
	0.696+	42	29	4.35	1.92	9.86	4.94	1.73	14.13
Day 4	< = 0.56	62	10	1.00	--		1.00	--	
	0.57+	40	27	4.19	1.83	9.57	4.81	1.72	13.44
Day 5	< = 0.28	53	12	1.00	--		1.00	--	
	0.29+	42	23	2.42	1.08	5.42	2.71	0.99	7.42
Day 6	< = 0.20	44	11	1.00	--		1.00	--	
	0.21+	34	19	2.24	0.94	5.32	2.10	0.63	7.04
		**With PGD Grade 3 (N = 28)**							
		**Infection**							
**PCT LEVELS**	**Cut-off point**	**NO (N)**	**YES (N)**	**ORc**^**b**^	**(95%**	**CI)**	**ORa**^**c**^	**(95%**	**CI)**
Admiss	<= .50	7	1	1.00	--		1.00	--	
	.51+	17	3	1.24	0.11	14.01	1.44	0.10	20.39
Day 1	< = 1.17	4	1	1.00	--		1.00	--	
	1.18+	20	3	0.60	0.05	7.35	1.10	0.07	16.55
Day 2	< = 1.14	6	1	1.00	--		1.00	--	
	1.15+	12	3	1.50	0.13	17.67	1.83	0.09	37.04
Day 3	< = 0.695	4	1	1.00	--		1.00	--	
	0.696+	6	1	0.67	0.03	14.03	--	--	--
Day 4	< = 0.56	2	1	1.00	--		1.00	--	
	0.57+	6	0	--	--	--	--	--	--
Day 5	< = 0.28	4	1	1.00	--		1.00	--	
	0.29+	5	0	--	--	--	--	--	--
Day 6	< = 0.20	2	1	1.00	--		1.00	--	
	0.21+	4	0	--	--	--	--	--	--

PGD = Primary Graft Dysfunction. (a) Levels categorized according to median. Levels < = cut-off point were taken as the reference category. (b) ORc denotes Crude OR. CI denotes confidence interval. (c) ORa refers to OR adjusted for sex, age (as continuous variable), necessity of post-operative Extra Corporeal Membrane Oxygenation (ECMO) (yes/no), creatinine levels (as continuous variable), type of lung transplant (single or double-lung transplantation) and trasplant centre (hospital).

## Discussion

Serum PCT levels are shown to be a good tool in diagnosing infectious complications after lung transplantation. However, their accuracy in detecting infections depends on the coexistence of severe PGD. CRP, meanwhile, was less useful than PCT in detecting postoperative complications after LT, irrespective of their cause.

In transplant recipients, PCT has been shown to be elevated during bacterial infection and to remain normal in the setting of acute rejection. However, most studies include mixed populations of solid organ transplant recipients, and only 2 have exclusively evaluated lung transplant recipients. In agreement with our results, both studies lung transplant recipients reported significantly higher peak PCT concentrations in patients with postoperative infection [15,16]. However, our earlier study did not evaluate PGD, while Desmard et al. did not evaluate the potential effect of PGD on the capacity of PCT to diagnose infection. The present study, which has the largest sample size to date, highlights the confounding effect of PGD grade 3 on the interpretation of PCT values. Therefore, although PCT did not detect subsequent development of infection in the subgroup of patients with PGD grade 3, it was shown to be a useful marker of infection in the absence of PGD. Thus, PCT levels higher than 0.50 ng/ml on ICU admission or 1.17 ng/ml on postoperative day 1 were associated with a three- and four-fold increase in risk of infection, respectively. These results support the potential utility of PCT in infection diagnosis in this setting. Although the pathogenesis of PGD is multifactorial and is still not fully understood, it seems to revolve around an ischemia-reperfusion phenomenon that causes a powerful inflammatory reaction. Certain cytokines, such as IL-6 and IL-8 are thought to be involved in the presentation and intensity of this clinical picture [17–20]. Similarly, PCT synthesis is closely linked to cytokine-mediated signalling [21,22], and its secretion is proportional to the magnitude of the underlying inflammatory process. This pathogenic nexus would justify the elevated levels of PCT observed in our study patients with PGD grade 3 and the insignificant elevation in patients with less severe dysfunction (probably due to insufficiently intense signalling). The small size of his series could have been a factor in Desmard’s failure to observe a correlation between PGD severity and PCT levels on postoperative day 1 [16].

The procalcitonin kinetic profile was similar for all patients, irrespective of the presence of complications or of their type. We observed an initial increase in PCT levels that peaked in the first 48 hours, followed by a progressive decline to < 0.5 ng/ml on postoperative day 7 in all cases. This is a common kinetic profile not only after lung transplantation and thoracic surgery, but also in other types of solid organ transplantation and in major surgery [23–28]. The difference between groups of patients, therefore, lies not in changes in PCT levels over time, but in the peak level observed. This is why PCT trends (delta value) are meaningless in this patient population. Even though Desmard et al. defend the utility of serial PCT determinations in these patients, they themselves acknowledge that the high inter-individual variability in PCT levels observed in the first postoperative week of their study limited the utility of this marker. Infection correlated with *de novo* elevation in PCT values after postoperative day 6, once levels had normalised [16]. The fact that PGD was not investigated as a confounding factor, even though it was observed in 35% of patients, could explain these results. In our study, PCT levels obtained in the first postoperative day in patients with no PGD grade 3 were significantly associated with a four-fold increase in the risk of infection, and were as reliable as those obtained on the following days. Bearing in mind that microbiological identification and data testing usually take at least 48 to 72 hours from the time the sample is obtained, PCT use could contribute useful information in these patients. Moreover, classic signs of infection, such as fever, may be masked by the use of steroids and other immunosuppressants, thus hindering diagnosis of infection. Most immunosuppressants, such as azathioprine, cyclosporine, tacrolimus, corticosteroids and anti-interleukin 2 monoclonal antibodies, do not to appear to interfere with PCT secretion [29–31], suggesting that it can be used in association with these drugs. Elevated PCT levels on the first postoperative day, therefore, should prompt clinicians to start thorough microbiological tests, and even to step up antibiotic therapy. A drug-escalation algorithm and intensified diagnostics based on daily PCT measurements in critically ill patients failed to improve survival [32]. For that reason, clinical trials focused specifically on this population are needed to confirm this hypothesis.

Regarding diagnostic accuracy, one of the problems with the test is the fact that the optimal cut-off point varies from day to day and so makes it difficult to use in practice, because of it we explored the strength of associations according to median split methods. PCT has so far been shown to be superior to CRP in the diagnosis of infection in the immediate postoperative period in transplant recipients. In this respect, most studies have focussed on liver and heart recipients [8,24]. Although data relating to postoperative follow-up in lung recipients is scant, and what little evidence is available is based on studies that have included both lung and heart transplant recipients, the results seems to point in the same direction [9,10,33]. Thus, Hammer et al. in a mixed population of heart transplant and lung transplant recipients found PCT levels to be predictors of infection, and moreover to be directly correlated with severity of infection [9,10]. In our previous study, albeit with a small sample size, PCT was shown to be superior to CRP and leukocyte determination in infection diagnosis during the first week after lung transplantation [15]. Our findings confirm the superiority of PCT over CRP in this context. Unlike PCT, CRP was not useful in diagnosing the presence of infection, irrespective of the presence or primary graft dysfunction. Furthermore, the use of CRP in postoperative follow-up of lung transplant patients seems to be unjustified.

The main strength of this study, aside from the sample size, is the heterogeneity of the patient population and its multicentre design. Seven hospitals across Spain, using different antibiotic prophylaxis and immunosuppressive regimens, and with different local microbial flora, participated in the trial. These characteristics support the potential external validity of our findings.

Our study has several limitations. First, we investigated only the first 7 postoperative days after lung transplantation. Our results, therefore, may not be valid for LT recipients developing infection beyond this time period. Secondly, donor PCT levels were unknown, and therefore early PCT measurements after LT could be influenced by donor secretion. Although little is known of the effect of donor secretion, Eyraud et al., studying a liver transplant population, showed high PCT peak levels in recipients to be associated with infection and cardiac arrest in donors. [34]. In our study, PCT peak levels were obtained 48 hours after LT; bearing in mind that the half-life of PCT is 24 hours, donor secretion is unlikely to have affected our measurements. Thirdly, we did not consider the immunosuppressive therapy used in our patients. However, as mentioned above, steroids and commonly used immunosuppressant drugs do not interfere with PCT metabolism. None of the study centres used OKT-3, the only immunosuppressant known to interfere with PCT production and secretion [35]. Finally, the biomarkers were measured in real time and the results were available to the clinicians treating the patients. It introduces a possibility of bias as the clinicians might rely on the biomarker for the diagnosis of infection and PGD. In all cases investigators were encouraged to adhere to International Society for Heart and Lung Transplantation definitions but the real time design of this study makes this bias possible.

## Conclusions

In the absence of severe PGD, PCT peak levels are useful in detecting the development of infectious complications during the first postoperative week. PGD grade 3 significantly increases PCT levels and interferes with the capacity of PCT as a marker of infection. PCT was superior to CRP in the diagnosis of infection during the study period.

## Supporting information

S1 Table Study population in relation to existence of complications (‘Infection’ or ‘primary graft dysfunction’). (DOCX)

S2 Table Descriptive of days between transplant performance and diagnosis of infection in transplant recipients. (DOCX)

S3 Table Valid cases and descriptive statistics for PCT and CRP levels in each day of follow-up, with respect to the existence of infection and primary graft dysfunction (PGD) grade 3. (DOCX)
