# Supplementary material for: Role of biomarkers in early infectious complications after lung transplantation
Source: PLoS One. 2017 Jul 13;12(7):e0180202. doi: 10.1371/journal.pone.0180202 (PMC5509107; doi:10.1371/journal.pone.0180202)
Supplement: S1 Table — (DOCX) [file pone.0180202.s001.docx]

**Supplementary table 1**. Study population in relation to existence of complications (‘Infection’ or ‘Primary graft dysfunction’)

| **Existence of Complications** | | | **Infection in trasplant recipient** | | **Total** |
| --- | --- | --- | --- | --- | --- |
|  |  |  | NO | YES |  |
| **No Complications** | Primary Graft Dysfunction | NO | 157 | ---- | 157 |
| **Only Infection** | Primary Graft Dysfunction | NO | ---- | 48 | 48 |
| **Only Dysfunction (Grade 3)** | Primary Graft Dysfunction | YES | 24 | ---- | 24 |
| **Infection and Dysfunction** | Primary Graft Dysfunction | YES | ---- | 4 | 4 |
| **Total** | Primary Graft Dysfunction | NO | 157 | 48 | 205 |
|  |  | YES | 24 | 4 | 28 |
|  | Total | | 181 | 52 | 233 |
