# Supplementary material for: Role of biomarkers in early infectious complications after lung transplantation
Source: PLoS One. 2017 Jul 13;12(7):e0180202. doi: 10.1371/journal.pone.0180202 (PMC5509107; doi:10.1371/journal.pone.0180202)
Supplement: S2 Table — (DOCX) [file pone.0180202.s002.docx]

**Supplementary table 2**. Descriptive of days between transplant performance and diagnosis of infection in transplant recipients

| **Day of ‘diagnosis of infection’ in transplant recipient** | | |
| --- | --- | --- |
| Day of ICU Admission (Day 0), n (%) | 18 | 34.6% |
| Day 1, n (%) | 9 | 17.3% |
| Day 2, n (%) | 3 | 5.8% |
| Day 3, n (%) | 5 | 9.6% |
| Day 4, n (%) | 6 | 11.5% |
| Day 5, n (%) | 6 | 11.5% |
| Day 6, n (%) | 5 | 9.6% |
| Total, n (%) | 52 | 100.0% |
| Days, Mean [SD] | 2.23 | 2.26 |
| Days, Median (IQR) | 1.00 | 0-4 |

SD=Standard Deviation. IQR=Interquartile Range.
