# Supplementary material for: Role of biomarkers in early infectious complications after lung transplantation
Source: PLoS One. 2017 Jul 13;12(7):e0180202. doi: 10.1371/journal.pone.0180202 (PMC5509107; doi:10.1371/journal.pone.0180202)
Supplement: S3 Table — (DOCX) [file pone.0180202.s003.docx]

**Supplementary Table 3**. Valid Cases and descriptive statistics for PCT and CRP levels in each day of follow-up, with respect to the existence of Infection and Primary Graft Dysfunction (PGD) Grade 3.

|  | **No Infection** |  |  |  |  | **Infection in trasplant recipient** |  |  |  |  | **No PGD Grade 3** |  |  |  |  | **PGD Grade 3** |  |  |  |  |
| --- | --- | --- | --- | --- | --- | --- | --- | --- | --- | --- | --- | --- | --- | --- | --- | --- | --- | --- | --- | --- |
|  | **N=181** |  |  |  |  | **N=52** |  |  |  |  | **N=205** |  |  |  |  | **N=28** |  |  |  |  |
| **PCT LEVELS** | **Valid** | **Missing^a^** | **Median** | **P25** | **P75** | **Valid** | **Missing** | **Median** | **P25** | **P25** | **Valid** | **Missing** | **Median** | **P25** | **P75** | **Valid** | **Missing** | **Median** | **P25** | **P75** |
| Admiss | 175 | 6 | 0.36 | 0.05 | 1.86 | 47 | 5 | 2.00 | 0.32 | 7.45 | 194 | 11 | 0.47 | 0.05 | 1.93 | 28 | 0 | 4.57 | 0.25 | 11.82 |
| Day 1 | 176 | 5 | 1.01 | 0.30 | 2.81 | 49 | 3 | 3.83 | 0.90 | 8.90 | 197 | 8 | 1.07 | 0.32 | 2.97 | 28 | 0 | 4.90 | 1.46 | 14.16 |
| Day 2 | 149 | 32 | 0.94 | 0.47 | 2.00 | 48 | 4 | 2.73 | 0.83 | 6.70 | 175 | 30 | 1.01 | 0.43 | 2.47 | 22 | 6 | 2.61 | 0.84 | 9.31 |
| Day 3 | 115 | 66 | 0.55 | 0.21 | 1.40 | 41 | 11 | 1.60 | 0.58 | 4.13 | 144 | 61 | 0.69 | 0.27 | 1.80 | 12 | 16 | 1.32 | 0.31 | 5.43 |
| Day 4 | 110 | 71 | 0.50 | 0.18 | 0.97 | 38 | 14 | 0.78 | 0.38 | 2.68 | 139 | 66 | 0.54 | 0.18 | 1.10 | 9 | 19 | 1.11 | 0.27 | 2.53 |
| Day 5 | 104 | 77 | 0.20 | 0.09 | 0.60 | 36 | 16 | 0.40 | 0.19 | 1.09 | 130 | 75 | 0.28 | 0.10 | 0.62 | 10 | 18 | 0.48 | 0.10 | 1.23 |
| Day 6 | 84 | 97 | 0.20 | 0.09 | 0.40 | 31 | 21 | 0.30 | 0.13 | 0.80 | 108 | 97 | 0.20 | 0.10 | 0.40 | 7 | 21 | 0.39 | 0.10 | 0.66 |
| **CRP LEVELS** | **Valid** | **Missing** | **Median** | **P25** | **P75** | **Valid** | **Missing** | **Median** | **P25** | **P75** | **Valid** | **Missing** | **Median** | **P25** | **P75** | **Valid** | **Missing** | **Median** | **P25** | **P75** |
| Admiss | 165 | 16 | 4.29 | 1.10 | 9.82 | 46 | 6 | 4.34 | 1.29 | 11.95 | 186 | 19 | 4.00 | 1.14 | 9.08 | 25 | 3 | 5.64 | 1.13 | 13.08 |
| Day 1 | 164 | 17 | 12.25 | 7.21 | 16.53 | 48 | 4 | 12.90 | 7.31 | 18.20 | 187 | 18 | 12.90 | 7.20 | 16.84 | 25 | 3 | 11.40 | 7.69 | 16.43 |
| Day 2 | 139 | 42 | 9.80 | 5.10 | 14.62 | 42 | 10 | 10.90 | 6.55 | 17.03 | 168 | 37 | 10.00 | 5.36 | 14.62 | 13 | 15 | 12.50 | 5.50 | 22.13 |
| Day 3 | 121 | 60 | 5.76 | 2.60 | 10.41 | 45 | 7 | 6.60 | 3.40 | 13.18 | 152 | 53 | 5.72 | 2.70 | 10.55 | 14 | 14 | 8.46 | 2.51 | 13.65 |
| Day 4 | 103 | 78 | 3.80 | 1.60 | 7.60 | 37 | 15 | 4.90 | 2.65 | 10.69 | 127 | 78 | 3.99 | 1.65 | 7.75 | 13 | 15 | 5.70 | 3.65 | 11.94 |
| Day 5 | 92 | 89 | 2.55 | 1.40 | 5.94 | 33 | 19 | 4.10 | 2.18 | 9.13 | 112 | 93 | 2.62 | 1.40 | 6.15 | 13 | 15 | 5.40 | 3.97 | 7.76 |
| Day 6 | 70 | 111 | 2.42 | 0.90 | 6.68 | 24 | 28 | 2.16 | 1.28 | 7.07 | 87 | 118 | 2.10 | 1.00 | 6.57 | 7 | 21 | 5.54 | 3.31 | 13.11 |

^a^ Number of patients without determination for this day.
